# Memory impairment is not sufficient for choice blindness to occur

**DOI:** 10.3389/fpsyg.2014.00449

**Published:** 2014-05-20

**Authors:** Anna Sagana, Melanie Sauerland, Harald Merckelbach

**Affiliations:** Forensic Psychology Section, Department of Clinical Psychological Science, Faculty of Psychology and Neuroscience, Maastricht UniversityMaastricht, Netherlands

**Keywords:** choice blindness, memory impairment, forgetting, confidence, decision making

## Abstract

Choice blindness refers to the phenomenon that people can be easily misled about the choices they made in the recent past. The aim of this study was to explore the cognitive mechanisms underlying choice blindness. Specifically, we tested whether memory impairment may account for choice blindness. A total of *N* = 88 participants provided sympathy ratings on 10-point scales for 20 female faces. Subsequently, participants motivated some of their ratings. However, on three trials, they were presented with sympathy ratings that deviated from their original ratings by three full scale points. On nearly 41% of the trials, participants failed to detect (i.e., were blind) the manipulation. After a short interval, participants were informed that some trials had been manipulated and were asked to recall their original ratings. Participants adopted the manipulated outcome in only 3% of the trials. Furthermore, the extent to which the original ratings were accurately remembered was not higher for detected as compared with non-detected trials. From a theoretical point of view our findings indicate that memory impairment does not fully account for blindness phenomena.

## INTRODUCTION

Lay people often think that humans are permanently aware of the reasons that guide their decisions. In fact the ability to compare the outcome of our choices with our intentions is vital for adaptive behaviour (Ridderinkhof et al., 2004; Ullsperger and von Cramon, 2004). Convinced of the rationality of their actions, people have strong opinions about their preferences and decisions. However, recent findings indicate that people are poor at detecting deviations from their choices. More specifically, a phenomenon named *choice blindness* demonstrates that people often fail to detect a mismatch between their intention and the later outcome when their choice is surreptitiously manipulated. In the original demonstration of the phenomenon (Johansson et al., 2005), participants had to select the more attractive alternative out of 15 different pairs of female faces. Subsequently, they were presented with the chosen alternative and were asked to explain their judgement. However, for three of these pairs the researchers, using a magic card trick, swapped the chosen photo with the alternative non-preferred face. Thus, participants had to justify a decision they never made. Astonishingly, only 13% of the manipulated trials were detected immediately at the time of the manipulation.

The basic phenomenon of choice blindness has been widely replicated (e.g., Johansson et al., 2008; Hall et al., 2010; Sauerland et al., 2013a) in different domains that are highly relevant to everyday decision making, such as consumer preferences (Hall et al., 2010), moral decision making (Hall et al., 2012), and symptom reports (e.g., Merckelbach et al., 2011). Choice blindness manipulations have been shown to result in changes of inclination toward a specific product and moral attitude, and in symptom escalation.

Yet little is known about the mechanism underlying the choice blindness phenomenon. Previous studies have successfully ruled out a number of possible explanations, including poor encoding of the original (Johansson et al., 2005) or manipulated stimuli (Sauerland et al., 2013a), compliance (Johansson et al., 2005, 2008; Sauerland et al., 2013a), suggestibility or the tendency to react in socially desirable ways (Merckelbach et al., 2011; Sauerland et al., 2013a, b). However, none of these studies specifically aimed at tackling the cognitive mechanisms underlying the phenomenon. The present work is a first, but crucial step to fill in this gap.

Memory distortion seems intuitively the most plausible candidate to explain choice blindness phenomena. Memory distortion is closely related to the misinformation effect (Loftus and Hoffman, 1989) and hindsight bias (Fischhoff, 1975) and in these domains, it possesses empirical merits. Interestingly, methods employed to study the misinformation effect and hindsight bias resemble the choice blindness paradigm. Specifically, all three involve exposure to an original stimulus, the subsequent confrontation with follow-up information (that may be misleading), and a final instruction that elicits the phenomenon (“Was there a stop sign?” “For which president candidate did you vote?” or “Why did you find this face the most attractive?”). Thus, all three phenomena can be conceptualized as forms of memory distortion caused by new information that influences a memory trace created earlier. Hence, it is reasonable to anticipate that the mechanisms underlying these phenomena share certain commonalities.

To explain misinformation and hindsight bias, researchers introduced the *memory impairment framework* (Fischhoff, 1975; Loftus and Hoffman, 1989). According to this framework, the original memory trace is permanently distorted by the presentation of new information by means of alteration, erasure or a decrease in accessibility of the original memory trace. Accordingly, exposure to follow-up information can interfere with the recollection of the original trace, hence resulting in memory impairment. Extreme positions within this framework hold that the new information blends with the existing trace, resulting in an imminent and persistent alteration of the original trace (immediate assimilation hypothesis; Fischhoff, 1975). More conventional approaches postulate that the retrieval of separate memory traces depends on the recency and depth of the encoding (dual memory traces theory; Hell et al., 1988) or available congruent information (selective retrieval hypothesis; Slovic and Fischhoff, 1977; Morton et al., 1985). Nonetheless, all accounts have in common that they view the interference of the new information as a cause of people’s inability to recall the original trace (Mazzoni and Vannucci, 2007). Thus, memory deterioration results from the failure to access the original memory trace.

Although memory deterioration is an intuitively plausible candidate to explain choice blindness, to our knowledge, there are no studies to date that have explicitly tested the memory-choice blindness link. Indirect evidence for such link comes from a field study (Sagana et al., 2013) that examined choice blindness for eyewitnesses’ facial recognition decisions. Sagana et al. (2013) reported that participants who made an accurate lineup decision were more likely to notice a covertly performed manipulation at the end of the experiment (i.e., *retrospectively*) than participants who made an erroneous recognition decision. In other words, participants’ memory, as indicated by recognition accuracy, was associated with higher detection rates. Although this study did not provide a direct test of memory strength, these findings are broadly consistent with the idea that memory deterioration is responsible for blindness phenomena. On the other hand, participants who immediately noticed the change (i.e., *concurrently*) were *not* more accurate in their lineup decisions than participants who were blind to the change. This suggests that the accuracy of a recognition decision and the capacity to detect a manipulation are not always associated. Thus, Sagana et al. (2013) concluded that blindness phenomena cannot be fully attributed to memory decay. The observation that blindness phenomena can be obtained even when the manipulated outcome is presented minutes after the original choice (Johansson et al., 2005; Hall et al., 2012) supports this position. Moreover, choice blindness may occur for stimuli of personal and moral significance (Hall et al., 2013; Sauerland et al., 2013b), again suggesting that memory decay may not be the sole mechanism driving the effect.

A direct test of whether memory impairment is a prerequisite for in choice blindness phenomena has not been done to date. This is the aim of the current study. To this end, we asked participants to rate the sympathy of female faces and secretly manipulated some of the judgments by increasing or decreasing their ratings. Shortly hereafter, participants were informed that some trials had been manipulated and they were asked to recall 50% of the original sympathy ratings they had made earlier. Given the common association between confidence and accuracy across different domains (Brewer and Wells, 2006; Rolls et al., 2010; Yeung and Summerfield, 2012), we also asked participants to provide a confidence rating.

Our research approach allowed us not only to measure memory strength, but also whether participants could sufficiently disentangle the original choice from the manipulated outcome once they had been notified about the manipulations. However, as we used a relatively short interval between the original ratings and the instruction to recall, one could argue that this is a limitation of the current a study. Specifically, one might argue that a relatively short interval may be suboptimal for studying memory corruption. However, given that participants were asked to recall ten different sympathy ratings of unfamiliar faces, we do think that we were able to tap into memory processes. Conversely, if a longer interval would have been introduced, then our results would run the risk of being burdened by a disproportionate increase in cognitive load (Klimesch et al., 1993). Importantly, misleading post-event suggestions can impair memory with short retention intervals (Belli et al., 1994). A second feature of our approach is that, on the second round of trials, we asked participants to recall their evaluations of the faces. Asking participants to recall their original ratings may be problematic, because, instead of recalling their original rating, participants may simply reevaluate the faces, as if performing the task a second time. Hence, consistency with the original choice may not reflect genuine recall, but a consistency in preference. This is a valid concern, as we have no means of knowing whether participants indeed recalled or reevaluated. However, recent findings indicate that choice blindness manipulations can shape preferences in favor of the manipulated outcome in a second round of choices (i.e., reevaluation; Johansson et al., 2008, 2013; Sauerland et al., 2013b). Importantly, such a shift was evident for both blind participants and detectors. Hence, we can make predictions about the outcomes for the reported ratings for cases where participants (a) recalled or reevaluated, (b) memory is or is not impaired, and (c) participants are blind or detectors (see **Table 1**). For example, if our participants simply reevaluated the faces, a shift in their answers in favor of the manipulated outcome should be expected, regardless of memory impairment being a valid explanation for choice blindness and participants being blind or detectors. If, however, participants recall – as they were instructed – then blind participants and detectors should either differ in the reported ratings (i.e., impaired memory) or should both favor the original rating (i.e., not-impaired memory).

**Table 1:** Hypothetical outcomes for reported ratings given the cognitive process, the memory capacity and participants’ manipulation status.

Process	Memory	Manipulation status	Reported rating
Recall	Impaired	Blind	Manipulated
		Detectors	Original
	Not-impaired	Blind	Original
		Detectors	Original

Reevaluation	Impaired	Blind	Manipulated
		Detectors	Manipulated
	Not-impaired	Blind	Manipulated
		Detectors	Manipulated

To sum up, according to the memory impairment framework, the presentation of the manipulated outcome should hinder the recollection of the original rating. This view implies that participants will recall the original rating in the subsequent memory test more accurately for the non-manipulated compared to the manipulated trials. Additionally, participants aware of the change (i.e., detectors) are predicted to be able to discriminate between the two competing traces (original vs. manipulated) and thus be better able to recall the original rating than participants blind to the change. Furthermore, if the memory impairment framework is correct, blind participants will adopt the manipulated sympathy ratings as their own. Finally, detectors are predicted to display higher confidence in their ability to remember the original rating than blind participants.

## MATERIALS AND METHODS

### PARTICIPANTS

A total of *N* = 88 participants (27 men, *M*_age_ = 22.3 years, *SD*_age_ = 5.0, *age range*: 18–55) took part in the study. Most of them were undergraduate psychology students (90.9%), whereas the remaining participants (9.1%) had various professional backgrounds. Student participants received course credit in return for their participation, while for the non-academics no monetary or other incentives were granted. Participation was voluntary. All participants were naïve to the purpose of the study and were tested individually. The study was approved by the standing ethical committee of the faculty.

### STIMULI AND STIMULUS SELECTION

Twenty female facial photos were selected for the sympathy rating task. All faces were of British or Australian public features unknown to our participants and were extracted from a data base kindly provided by R. Jenkins (for full description of technical characteristics see, Jenkins et al., 2011). Photos showed the faces in roughly frontal aspect with neutral or smiling facial expression. Copyright restrictions prevent us from reproducing the images here. The size of the photos as presented on the computer screen was 4.5 × 6.5 cm and they were centered in the upper half of the screen.

To avoid our results being attributed to differences in distinctiveness of the manipulated photos, as the effect of this factor in choice blindness is not yet examined, we selected three photos to serve as the to-be-manipulated targets, from the aforementioned stimulus pool. These had been rated the least distinctive, the most distinctive and moderately distinctive in a pilot study with 18 participants (11 men, *M*_age_ = 25.6, *SD*_age_ = 8.31, *age range*: 19–48). These photos depicted Cilla Black (*M* = 3.9, *SD* = 2.4), Rachel Stevens (*M* = 4.9, *SD* = 1.7), and Carol Smilie (*M* = 5.9, *SD* = 1.7). Distinctiveness scores for their pictures differ from each other, all *t*s(17) ≥ 2.18, *p*s ≤ 0.058. However, detection did not vary as a function of distinctiveness, Wald *x*^2^s(1, *N* = 264) = 1.98, *p*s ≥ 0.376. We will therefore not discuss this factor any further.

### DESIGN

The dependent variables were consistency with the original rating and consistency with the manipulated outcome. Detection (blindness vs. detection) of the manipulated trials and confidence in the ability to recall the original sympathy ratings served as the independent variables. As a measure of detection we used participants immediate (i.e., concurrent) apprehension of a change in their sympathy ratings.

### PROCEDURE

All stimuli were presented on a computer screen at a resolution of 1024 × 768 pixels using *Open Sesame* display software (Mathôt et al., 2012). A cover story led participants to believe that the study was concerned with facial characteristics that make faces more or less sympathetic. The study consisted of two parts executed in a single session. During the first part, after signing the informed consent, participants were instructed to rate how sympathetic they found each of 20 female faces, using a scale ranging from 1 (*not sympathetic at all*) to 10 (*extremely sympathetic*). After a 2 s occlusion time, participants were presented with the picture and the corresponding sympathy rating again. For half of the presented faces (i.e., 10) participants were asked to briefly give reasons for their rating in written. For the remaining faces, participants simply had to press the space bar to continue. However, in three of the 10 trials where a justification was required, we increased or decreased participants’ original sympathy ratings by three full points. For each of the three trials, the manipulation was dependent on participants’ sympathy rating. Specifically, if participants provided a rating from 1 to 5, we increased their rating, but if participants provided a rating from 6 to 10, we decreased their initial rating. Detection was not affected by the direction of the manipulation, *x*^2^(1, *N* = 264) = 2.16, *p* = 0.143, φ = -0.09. If participants typed a comment indicating that the displayed rating did not correspond with their own or that the program had made a mistake, they were classified as detectors. Detectors frequently also verbally informed the experimenter about the change i.e., the “mistake.”

Following the completion of the sympathy rating task participants filled out personality questionnaires as a filler task. Subsequently, the second part of the study began, in which participants were informed that some of their sympathy judgments had been altered. No information was given as to which specific trials or how many of them had been manipulated. Participants’ task was to again view the 10 faces for which they had provided a motivation earlier and to recall their original sympathy ratings. Furthermore, participants indicated how confident they were that this corresponded with their original rating on an 11-point scale ranging from 0 to 100%. Subsequently, as in previous studies (e.g., Johansson et al., 2005; Hall et al., 2010; Sauerland et al., 2013a, b), participants received a questionnaire to determine whether they had detected the manipulations before we disclosed that information, but refrained from revealing it while performing the task. Given that many participants were confused by the structure of the questionnaire^1^, it is doubtful whether the trials reported in the post-test questionnaire reflect genuine detections. Therefore, we refrain from reporting the questionnaire data. Finally, participants were thanked and fully debriefed.

## RESULTS

### CHOICE BLINDNESS

Out of the 88 × 3 = 264 manipulated trials, 107 (40.5%) were detected. **Table 2** shows the detection rates for the three manipulated trials and across all 264 manipulated trials. One could argue that strictly speaking, choice reversal only occurs when manipulations involve changes crossing the mid-point of the rating scale (i.e., from “sympathetic” to “not sympathetic,” rather than from “sympathetic” to “less sympathetic”). Accordingly, we reran the analysis including only those participants, for which the manipulation resulted in the original choice being shifted to the opposite half of the Likert scale, leaving 221 trials for analysis. Ninety-one (41.6%) of these were detected and the pattern results for all subsequent analyses remained largely the same. Therefore, we report the analyses for the total sample.

**Table 2:** Proportion detection for the three manipulated trials.

	Detection	
	*M* (%)	95% CI
1st manipulation	35.2	25.0–45.0
2nd manipulation	39.8	29.5–50.0
3rd manipulation	46.6	43.2–64.7
Overall	40.5	38.8–47.0

### MEMORY IMPAIRMENT

To determine whether participants’ ability to remember the original ratings was impaired by the performed manipulations, we first compared memory for the sympathy ratings in manipulated vs. non-manipulated trials. For the non-manipulated trials, participants were able to exactly remember their original rating in 45.8% (278 of 607, 95% CI [42.0, 49.0]) of the trials. For the manipulated trials, that percentage was 46.2% (122 of 264, 95% CI [40.2, 52.3]). Evidently, there were no differences in participants ability to remember their original rating, *z* = -0.91, *p* = 0.91, indicating that blindness is not related to suboptimal memory.

**FIGURE 1:**
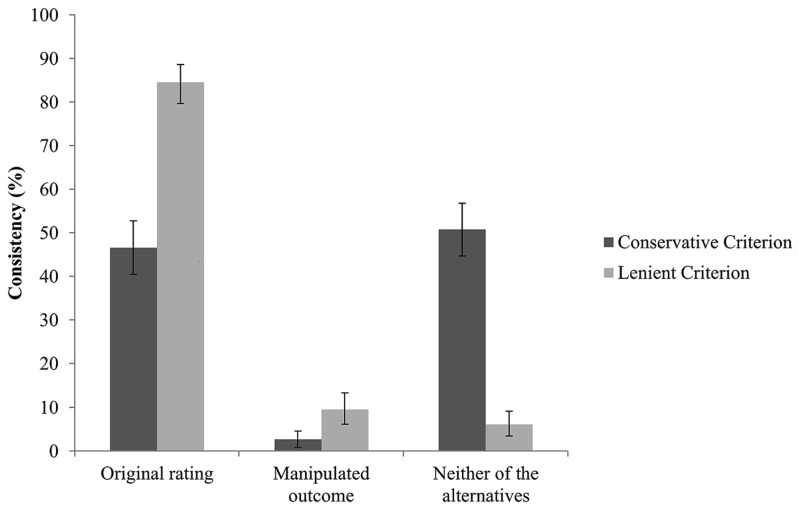
**Proportion of trials consistent with the original sympathy rating, the manipulated outcome, and neither of after the application of a conservative or lenient (±1 scale point variance) criterion**. Error bars represent 95% confidence intervals.

Next, we examined whether participants would adopt the manipulated sympathy ratings as their own as a result of impaired memory of the original choice. Comparisons of participants’ consistency with the manipulated outcome vs. consistency with the original rating revealed that participants were much more often consistent with their original rating than with the manipulated one, *McNemar x*^2^s (1, *N* = 264) = 101.73, *p* < 0.001. Specifically, across the 246 manipulated trials, participants reported the manipulated outcome in 2.7% of the trials (*SD* = 16.0, 95% CI [0.8, 4.9]), while participants were consistent with their original rating in 46.6% (*SD* = 49.9, 95% CI [40.2, 52.7]) of the trials. In the remaining trials (50.8%, *SD* = 50.1, 95% CI [44.3, 56.8]), participants were inconsistent with both the manipulated outcome and the original rating (see **Figure 1**). Applying a more liberal consistency criterion, of ±1 scale point^2^ as equivalent to the original rating or the manipulated outcome led to similar results, *McNemar x*^2^(1, *N* = 264) = 156.49, *p*s < 0.001. Specifically, 9.5% (*SD* = 29.3, 95% CI [6.1, 13.3]) of the 246 manipulated trials were similar to the manipulated outcome, while 84.5% (*SD* = 36.3, 95% CI [79.6, 88.6]) were consistent with the original rating, and 6.1% (*SD* = 23.9, 95% CI [3.4, 9.1]) with neither of the trials. Apparently, manipulating the original sympathy rating did not lead to overall memory distortion.

Next, we tested the hypothesis that consistency with the original rating varies as a function of detection. No support was found for this assumption, *x*^2^(2, *N* = 264) = 2.38, *p* = 0.122, φ = 0.09. Specifically, consistency with the original sympathy rating was met in 42.7% (*SD* = 49.6, 95% CI [35.1, 50.7]) of the non-detected and in 52.3 % (*SD* = 50.2, 95% CI [42.9, 61.7]) of the detected trials. Thus, memory impairment alone, in not a sufficient explanation for choice blindness phenomena.

**FIGURE 2:**
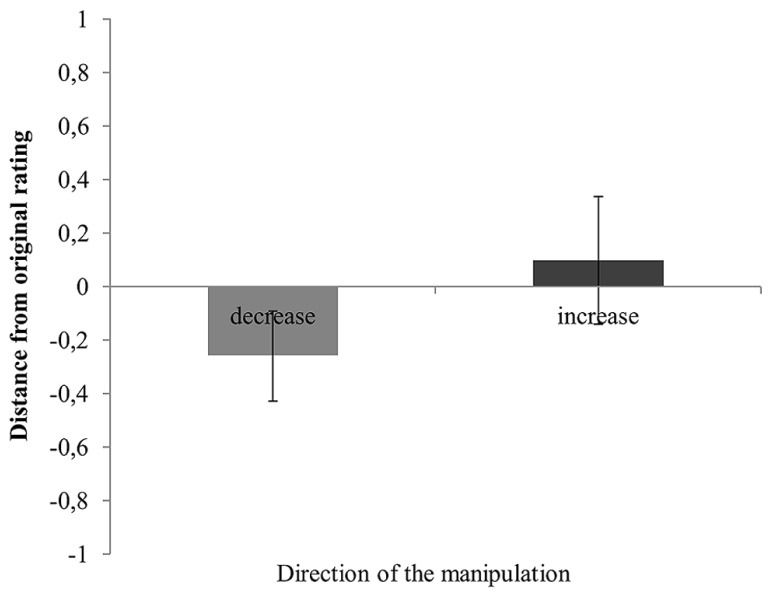
**Mean distance from the original sympathy rating as a function of manipulation direction (increase vs. decrease)**. Error bars represent 95% confidence intervals.

In light of these findings, we wondered whether our manipulations had any effect on participants recall ability. To test this, we computed the deviation between the remembered and participants’ original sympathy ratings (i.e., Consistency = rating time 2 – rating time 1) as a continuous measure of consistency with the original rating. Our aim was to examine whether this alternative approach would reveal differences in recall ability between manipulated and non-manipulated trials. Two outliers with a deviation of five scale points from the original choice were excluded. A paired sample *t*-test showed that the mean distance between the recalled and the original sympathy rating was greater for manipulated (*M* = 0.53, *SD* = 0.49, 95% CI [0.43, 0.64]) than non-manipulated trials (*M* = 0.27, *SD* = 0.22, 95% CI [0.22, 0.31]), *t*(85) = 4.59, *p* < 0.001. Additionally, the direction of the change from participants’ original to their remembered estimate was consistent with the direction of the manipulation (although it did not affect the distance between participants’ original and remembered estimate, *t*(260) = 1.75, *p* = 0.080). **Figure 2** displays the mean distance from the original sympathy ratings for increase (*n* = 123) and decrease (*n* = 141) manipulations. As can be seen, the manipulations affected participants’ recollections.

**FIGURE 3:**
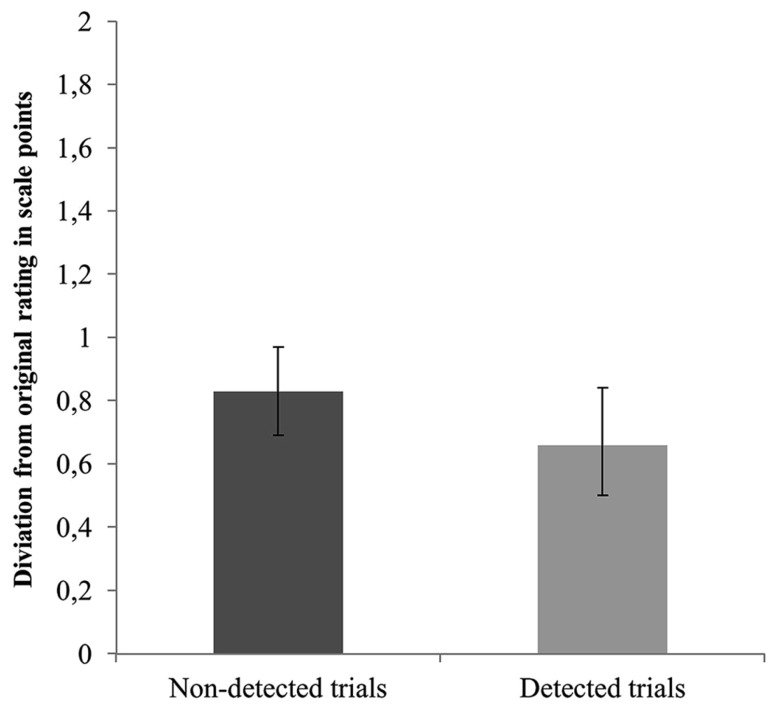
**Mean distance from the original sympathy rating as a function of detection (detected vs. non-detected)**. Error bars represent 95% confidence intervals.

Following a similar approach, we next examined whether this continuous measure of consistency would reveal differences in recall ability between detected and non-detected trials. Hence, we performed general equations estimates (GEE) analyses with consistency with the original sympathy estimate as dependent variable and detection (blindness vs. detection) as predictor. However, no significant results emerged, Wald *x*^2^(1, *N* = 262) = 2.7, *p* = 0.141. **Figure 3** displays the mean distance from the original sympathy ratings as a function of detection. Finally, we tested whether the distance from the original sympathy rating would be greater for the non-detected compared with the detected trials among the inconsistent with the original rating cases (*N* = 139). No support was found for this assumption, Wald *x*^2^(1, *N* = 139) = 0.20, *p* = 0.652. Specifically, the mean distance from the original sympathy rating for the non-detected trials was *M* = 1.45 (*SD* = 0.76, 95% CI [1.30, 1.63]) and for the detected trials was *M* = 1.39 (*SD* = 0.85, 95% CI [1.18, 1.63]). Evidently, memory impairment is not sufficient for choice blindness to occur.

### CONFIDENCE AS POSTDICTOR OF CONSISTENCY

**FIGURE 4:**
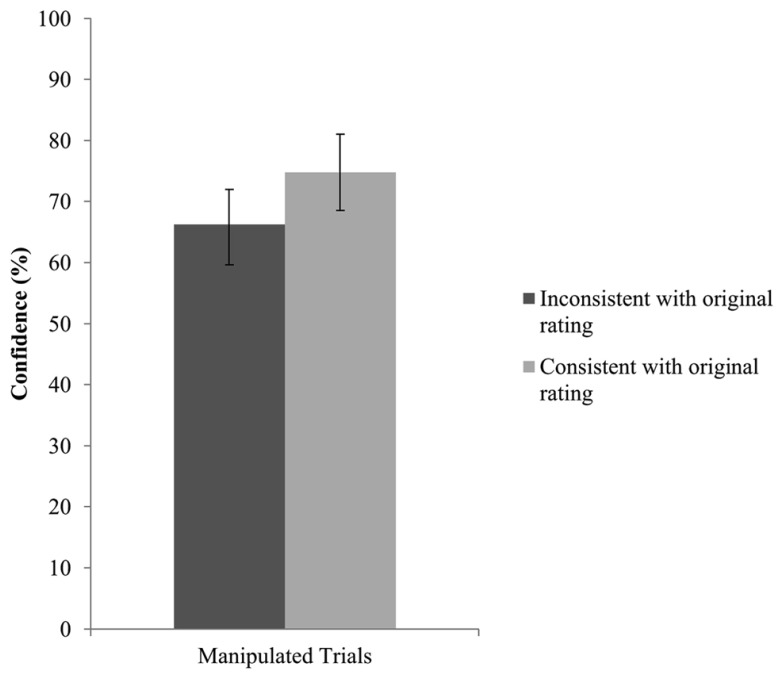
**Confidence as a function of consistency with the original sympathy rating for all manipulated trials**. Error bars represent 95% confidence intervals.

The final set of analyses tested whether confidence in recall ability, along with detection, can postdict consistency with the original sympathy rating. We performed GEE analyses with consistency with the original sympathy estimate as dependent variable and detection (blindness vs. detection) and confidence as predictors. In 14 trials, no confidence rating was provided leaving 250 trials for analysis. The interaction effect between detection and confidence was not significant, Wald *x*^2^(1, *N* = 250) = 2.80, *p* = 0.094. The model without the interaction term returned no significant effect for detection, Wald *x*^2^(1, *N* = 250) = 0.621, *p* = 0.431, but revealed a significant confidence effect. After deleting detection from the model, confidence attained significance, Wald *x*^2^(1, *N* = 250) = 9.33, *p* = 0.002. Specifically, higher confidence ratings were associated with higher consistency with the original choice. Thus, confidence seemed to be a reliable predictor of consistency with the original rating. **Figure 4** presents the confidence ratings for all manipulated trials that are consistent or inconsistent with the original rating.

**FIGURE 5:**
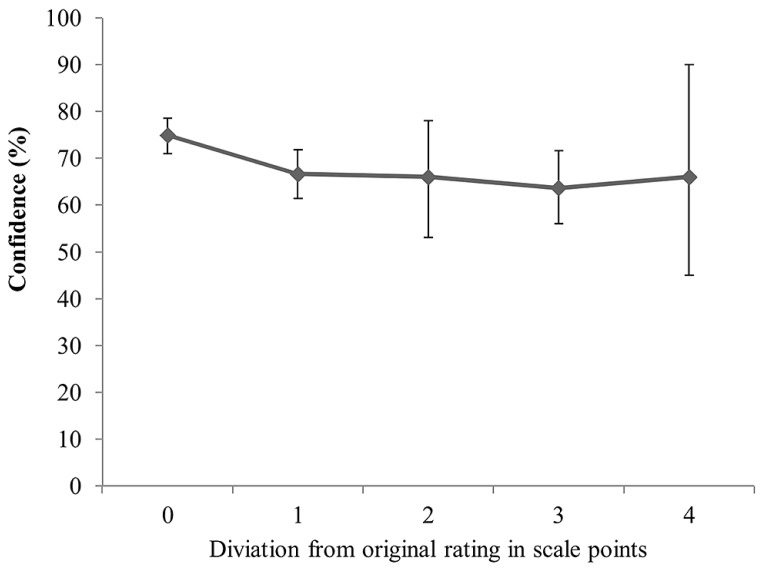
**Confidence as a function of the deviation from the original sympathy rating for detected and non-detected trials**. Error bars represent 95% confidence intervals.

Using the continuous measure of consistency led to comparable results. Specifically, neither the interaction between detection and confidence, Wald *x*^2^(1, *N* = 249) = 0.93, *p* = 0.334, nor the main effect of detection (after deletion of the interaction effect) was significant, Wald *x*^2^(1, *N* = 250) = 0.602, *p* = 0.438. The main effect of confidence indicated that high confidence was associated with little deviation from the original rating, while lower confidence was associated with stronger deviations from the original rating, Wald *x*^2^(1, *N* = 249) = 5.96, *p* = 0.015. **Figure 5** displays mean confidence across the observed distances from the original rating.

## DISCUSSION

The aim of the current study was to test whether choice blindness is a result of memory impairment of the original trace. To our knowledge, this is the first study that directly examined this issue. Drawing on the memory impairment framework (Fischhoff, 1975; Loftus and Hoffman, 1989), we predicted that participants would have difficulties in remembering their original sympathy rating after confrontation with a manipulated rating and that they would adopt the manipulated outcome as their own. Contrary to this assumption, the vast majority of the participants gave sympathy ratings that were consistent with their original choice. Further, there were no differences between detectors and blind participants in their ability to reproduce their original ratings. This was true when consistency with the original ratings was expressed in dichotomous terms, but also while being indexed in a continuous fashion. Although participants were affected by the direction of the manipulation, the distance between the recalled and the original sympathy rating did not differ among detected and non-detected trials. Furthermore, participants’ confidence in their ability to remember the original sympathy rating was a reliable postdictor of consistency.

Despite the fact that choice blindness can occur in the absence of memory impairment, the current study allows for other important observations. First, our results provide further evidence for the robustness of the choice blindness effect (e.g., Johansson et al., 2008; Hall et al., 2010; Sauerland et al., 2013a). Second, our findings are comparable with the broader misinformation literature which shows that *post hoc* misinformation often distorts memory (for a review, see Loftus and Hoffman, 1989; Payne et al., 1994; Ayers and Reder, 1998). Evidently, there were differences in the remembered sympathy rating between manipulated and non-manipulated trials and participants were largely affected by the direction of the manipulation (see **Figure 2**). The fact that consistency with the original sympathy rating was not associated with the capacity to detect a manipulation though, speaks to the idea that distorted memory is not necessary for choice blindness to occur.

Third, the current study is informative as it demonstrates with a direct test that blindness phenomena cannot be attributed to impaired memory of the initial memory trace or memory decay. Specifically, in a substantial number of trials (123 of 264; 46.6%), participants were able to remember their original rating with precision. This supports the idea that the memory trace for the original rating was still accessible and could be retrieved at will. These findings support earlier indications that blindness phenomena cannot be reduced to weak memory or forgetting (Sagana et al., 2013). Over and above when participants did change their ratings (53.4% of trials), the shift in the answers from the original sympathy ratings to the subsequently remembered sympathy estimates was independent of whether a manipulation was detected or not. Thus, even when participants were aware of the change in their judgments, they had limited capacity to segregate the influence of the manipulation from the recall process. This replicates earlier findings showing that participants shift their answers in favor of the originally non-preferred alternative when asked to perform a second round of choices (Johansson et al., 2008; Hall et al., 2013; Sauerland et al., 2013b). These studies and our findings are consistent with work demonstrating that there are conditions in which choices *precede* preferences (rather than vice versa; see studies by Sharot et al., 2009, 2010).

Further, our results demonstrate that confidence is a reliable postdictor of consistency with the original sympathy rating. Hence, the present findings indicate the informative value of confidence as a postdictor of the accuracy of estimates provided following a manipulated outcome; that is, following incorrect information about one’s own decision. Considering that to date we only have limited understanding of the mechanism contributing to choice blindness – and thus incapable of offering methods for its reversal – the postdictive value of confidence might be beneficial for establishing the accuracy of a remembered choice.

Turning to the limitations of the present study, as already discussed in the introduction participants may not have engaged actively in retrieval, but rather may have provided a sympathy estimate as if they were asked to perform the same task a second time. However, the fact that both blind participants and detectors were consistent with the original rating speaks to the idea that participants, indeed, recalled their original rating instead of simply performing the task a second time (see **Table 1**). Furthermore, our procedure was largely parallel with that is typically done to evaluate memory for past decisions in hindsight bias literature (e.g., Hoffrage et al., 2000; Sanna and Schwarz, 2003; Pohl, 2007). Nevertheless, the contributions of retrieval vs. preferential consistency should be addressed more directly in future research. Another limitation is that the post-test questionnaire was handed out at the end of the experiment, after participants were informed about the manipulations. This made it difficult to derive retrospective detection rates. Additionally, the absence of the actual stimulus photos in the post-test questionnaire was a source of confusion. Future research should consider administering the post-test questionnaire before the recall task and including photos of the stimuli instead of verbal descriptions.

To summarize, this study has shown that memory impairment is not sufficient prerequisite for choice blindness to occur. Instead, our findings indicate a complex mechanism responsible for choice blindness that allows a fairly accurate recollection of the original choice but yet disables the detection of a manipulation. This arguably hints in the direction of a subtle malfunction in recognition. One way to test this interpretation is to examine differences in neuronal activity while participants perform a typical choice blindness paradigm and a subsequent recognition task. We acknowledge though that developing a choice blindness paradigm which permits the collection of neural data may be difficult, given that fMRI and ERP measures require a number of repetitive trials which in itself may introduce artefacts. An alternative explanation, however, is that participants are involved in a self-persuasion process (Johansson et al., 2011). Endorsing choices suggested by others may generate a degree of cognitive dissonance, which people want to overcome (Festinger, 1962; Henkel and Mather, 2007). Therefore, participants confabulate introspective arguments to convince themselves about the assumed choice. Hence, they fail to detect the manipulated outcome but are able to recall their original choice when the dissonance is resolved by the revelation that the choice had been manipulated.

## CONCLUSION

We believe that the results provide interesting insights for understanding choice blindness, with the major finding being that forgetting is not an exhaustive explanation for the phenomenon. Hence, to grasp the fundamentals of choice blindness, we may better consider Baddeley’s (1988) question: “What the hell is it for?” Therefore, we suggest that future research should focus on the function of blindness phenomena when answering the question about why they occur at all in everyday life. 

## Conflict of Interest Statement

The authors declare that the research was conducted in the absence of any commercial or financial relationships that could be construed as a potential conflict of interest.

## Footnotes

1 Participants were asked whether they had detected the manipulation and if so, they had to specify how many times the manipulation had occurred and which trials exactly had been manipulated. Unfortunately, we did not include the actual photos in the post-test questionnaire, an omission that caused confusion in the participants about which trial referred to which picture. However, only an additional 19 (7.2%) of the 264 trials were accurately reported in the post-test questionnaire raising the total detection rate to 47.7% and the pattern of results remained largely the same as for concurrent detection.

2 This is equal to roughly one SD difference in consistency with the original rating (*SD* = 0.89) and the manipulated choice (*SD* = 1.07).

## References

[B1] Ayers M. S. Reder L. M. (1998). A theoretical review of the misinformation effect: predictions from an activation-based memory model. *Psychon. Bull. Rev.* 5 1–21 10.3758/bf03209454

[B2] Baddeley A. (1988). “But what the hell is it for?” in *Memory in Everyday Life* Vol.1 *Practical Aspects of Memory: Current Research and Issues* edsGruneberg M. M. Morris P. E. Sykes R. N. (Chichester: John Wiley) 3–18

[B3] Belli R. F. Lindsay D. S. Gales M. S. Mccarthy T. T. (1994). Memory impairment and source misattribution in postevent misinformation experiments with short retention intervals. *Mem. Cognit.* 22 40–54 10.3758/BF03202760 8035684

[B4] Brewer N. Wells G. L. (2006). The confidence-accuracy relationship in eyewitness identification: effects of lineup instructions, foil similarity, and target-absent base rates. *J. Exp. Psychol. Appl.* 12 11–30 10.1037/1076-898X.12.1.11 16536656

[B5] Festinger L. (1962). *A Theory of Cognitive Dissonance*. Palo Alto, CA: Stanford University Press

[B6] Fischhoff B. (1975). Hindsight is not equal to foresight: the effect of outcome knowledge on judgment under uncertainty. *J. Exp. Psychol. Hum. Percept. Perform.* 1 288–299 10.1037/0096-1523.1.3.288 PMC1743746 12897366

[B7] Hall L. Johansson P. Strandberg T. (2012). Lifting the veil of morality: choice blindness and attitude reversals on a self-transforming survey. *PLoS ONE* 7:e45457 10.1371/journal.pone.0045457 PMC3446893 23029020

[B8] Hall L. Johansson P. Tärning B. Sikström, S. Deutgen T. (2010). Magic at the marketplace: choice blindness for the taste of jam and the smell of tea. *Cognition* 117 54–61 10.1016/j.cognition.2010.06.010 20637455

[B9] Hall L. Strandberg T. Pärnamets P. Lind A. Tärning B. Johansson P. (2013). How the polls can be both spot on and dead wrong: using choice blindness to shift political attitudes and voter intentions. *PLoS ONE* 8:e60554 10.1371/journal.pone.0060554 PMC3622694 23593244

[B10] Hell W. Gigerenzer G. Gauggel S. Mall M Müller M. (1988). Hindsight bias: an interaction of automatic and motivational factors? *Mem. Cognit.* 16 533–538 10.3758/bf03197054 3193884

[B11] Henkel L. A. Mather M. (2007). Memory attributions for choices: how beliefs shape our memories. *J. Mem. Lang.* 57 163–176 10.1016/j.jml.2006.08.012

[B12] Hoffrage U. Hertwig R. Gigerenzer G. (2000). Hindsight bias: a by-product of knowledge updating? *J. Exp. Psychol. Learn. Mem. Cogn.* 26 566–581 10.1037/0278-7393.26.3.566 10855418

[B13] Jenkins R. White D. Van Montfort X Mike Burton A. (2011). Variability in photos of the same face. *Cognition* 121 313–323 10.1016/j.cognition.2011.08.001 21890124

[B14] Johansson P. Hall L. Gardenfors P. (2011). Choice blindness and the non-unitary nature of the human mind. *Behav. Brain Sci.* 34 28–29 10.1017/S0140525x10002591

[B15] Johansson P. Hall L Sikström S. (2008). From change blindness to choice blindness. *Psychologia* 51 142–155 10.2117/psysoc.2008.142

[B16] Johansson P. Hall L. Sikström S. Olsson A. (2005). Failure to detect mismatches between intention and outcome in a simple decision task. *Science* 310 116–119 10.1126/science.1111709 16210542

[B17] Johansson P. Hall L. Tärning B. Sikström S. Chater N. (2013). Choice blindness and preference change: you will like this paper better if you (believe you) chose to read it! *J. Behav. Decis. Mak.* 10.1002/bdm.1807 [Epub ahead of print]

[B18] Klimesch W. Schimke H. Pfurtscheller G. (1993). Alpha frequency, cognitive load and memory performance. *Brain Topogr.* 5 241–251 10.1007/BF01128991 8507550

[B19] Loftus E. F. Hoffman H. G. (1989). Misinformation and memory: the creation of new memories. *J. Exp. Psychol. Gen.* 118 100–104 10.1037/0096-3445.118.1.100 2522502

[B20] Mathôt S. Schreij D. Theeuwes J. (2012). OpenSesame: an open-source, graphical experiment builder for the social sciences. *Behav. Res. Methods* 44 314–324 10.3758/s13428-011-0168-7 22083660 PMC3356517

[B21] Mazzoni G. Vannucci M. (2007). Hindsight bias, the misinformation effect, and false autobiographical memories. *Soc. Cogn.* 25 203–220 10.1521/soco.2007.25.1.203

[B22] Merckelbach H. Jelicic M. Pieters M. (2011). The residual effect of feigning: how intentional faking may evolve into a less conscious form of symptom reporting. *J. Clin. Exp. Neuropsychol.* 33 131–139 10.1080/13803395.2010.495055 20623399

[B23] Morton J. Hammersley R. H. Bekerian D. A. (1985). Headed records: a model for memory and its failures. *Cognition* 20 1–23 10.1016/0010-0277(85)90002-2 4017518

[B24] Payne D. G. Toglia M. P. Anastasi J. S. (1994). Recognition performance level and the magnitude of the misinformation effect in eyewitness memory. *Psychon. Bull. Rev.* 1 376–382 10.3758/bf03213978 24203521

[B25] Pohl R. F. (2007). Ways to assess hindsight bias. *Soc. Cogn.* 25 14–31 10.1521/soco.2007.25.1.14

[B26] Ridderinkhof K. R. Ullsperger M. Crone E. A. Nieuwenhuis S. (2004). The role of the medial frontal cortex in cognitive control. *Science* 306 443–447 10.1126/science.1100301 15486290

[B27] Rolls E. T. Grabenhorst F. Deco G. (2010). Choice, difficulty, and confidence in the brain. *Neuroimage* 53 694–706 10.1016/j.neuroimage.2010.06.073 20615471

[B28] Sagana A. Sauerland M. Merckelbach H. (2013). Witnesses’ blindness for their own facial recognition decisions: a field study. *Behav. Sci. Law* 31 624–636 10.1002/bsl.2082 24019073

[B29] Sanna L. J. Schwarz N. (2003). Debiasing the hindsight bias: the role of accessibility experiences and (mis)attributions. *J. Exp. Soc. Psychol.* 39 287–295 10.1016/S0022-1031(02)00528-0

[B30] Sauerland M. Sagana A. Otgaar H. (2013a). Theoretical and legal issues related to choice blindness for voices. *Legal Criminol. Psych.* 18 371–381 10.1111/j.2044-8333.2012.02049.x

[B31] Sauerland M. Schell J. Collaris J. Reimer N. Schneider M. Merckelbach H. (2013b). “Yes, I have sometimes stolen bikes”: blindness for norm-violating behaviors and implications for suspect interrogations. *Behav. Sci. Law* 31 239–55 10.1002/bsl.2063 23625799

[B32] Sharot T. Martino B. D. Dolan R. J. (2009). How choice reveals and shapes expected hedonic outcome. *J. Neurosci.* 29 3760–3765 10.1523/jneurosci.4972-08.2009 19321772 PMC2675705

[B33] Sharot T. Velasquez C. M. Dolan R. J. (2010). Do decisions shape preference? Evidence from bind choice. *Psychol. Sci.* 21 1231–1235 10.1177/0956797610379235 20679522 PMC3196841

[B34] Slovic P. Fischhoff B. (1977). On the psychology of experimental surprises. *J. Exp. Psychol. Hum. Percept. Perform.* 3 544–551 10.1037/0096-1523.3.4.544

[B35] Ullsperger M von Cramon D. Y. (2004). Neuroimaging of performance monitoring: error detection and beyond. *Cortex* 40 593–604 10.1016/S0010-9452(08)70155-2 15505969

[B36] Yeung N. Summerfield C. (2012). Metacognition in human decision-making: confidence and error monitoring. *Philos. Trans. R. Soc. Lond. B Biol. Sci.* 367 1310–1321 10.1098/rstb.2011.0416 22492749 PMC3318764

